# Hairy Root Cultures—A Versatile Tool With Multiple Applications

**DOI:** 10.3389/fpls.2020.00033

**Published:** 2020-03-03

**Authors:** Noemi Gutierrez-Valdes, Suvi T. Häkkinen, Camille Lemasson, Marina Guillet, Kirsi-Marja Oksman-Caldentey, Anneli Ritala, Florian Cardon

**Affiliations:** ^1^VTT Technical Research Centre of Finland Ltd., Espoo, Finland; ^2^Samabriva SA, Amiens, France

**Keywords:** *Rhizobium rhizogenes*, *Agrobacterium rhizogenes*, hairy roots, recombinant proteins, specialized metabolites, molecular farming

## Abstract

Hairy roots derived from the infection of a plant by *Rhizobium rhizogenes (previously referred to as Agrobacterium rhizogenes*) bacteria, can be obtained from a wide variety of plants and allow the production of highly diverse molecules. Hairy roots are able to produce and secrete complex active glycoproteins from a large spectrum of organisms. They are also adequate to express plant natural biosynthesis pathways required to produce specialized metabolites and can benefit from the new genetic tools available to facilitate an optimized production of tailor-made molecules. This adaptability has positioned hairy root platforms as major biotechnological tools. Researchers and industries have contributed to their advancement, which represents new alternatives from classical systems to produce complex molecules. Now these expression systems are ready to be used by different industries like pharmaceutical, cosmetics, and food sectors due to the development of fully controlled large-scale bioreactors. This review aims to describe the evolution of hairy root generation and culture methods and to highlight the possibilities offered by hairy roots in terms of feasibility and perspectives.

## Introduction

Between the 1930s and the 1960s, hairy roots (HRs) were studied primarily as a sign of pathogen invasion in horticultural plants (Doran, 2013). Only until the 1970s to the 1980s, *Agrobacterium rhizogenes* was identified as the bacterial agent that, through the gene transfer of the bacterial Ri (root-inducing) plasmid, induces HR syndrome (Chilton et al., 1982; Gelvin, 2009). This important revelation prompted several studies that have helped to develop the hairy root cultures (HRCs) technology. We now know that HRs arise from the wounding site of plantlets when they are infected by *A. rhizogenes*, a symbiotic bacterium currently taxonomically renamed *Rhizobium rhizogenes*. The infection takes place upon of specific bacterial DNA fragments (T-DNA) from its root-inducing plasmid (pRi) into plant cells (Chilton et al., 1982). Even when the plant responds to bacterial infection by triggering the expression of several defense-related proteins to suppress the pathogen, *R. rhizogenes* has evolved mechanisms to take advantage of those plant defense proteins by counteracting an action that consequently dismounts the plant defense pathways (Sevón and Oksman-Caldentey, 2002; Mauro et al., 2017). To date, every system for recombinant protein production presents its limitations. For instance, inability to produce and/or secrete functional complex proteins (e.g. bacterial systems), risk of viral transmission and toxic molecules (e.g. bacterial systems, mammalian cells), or very high production costs (e.g. mammalian cells) (Cardon et al., 2019). Since the early 2000s, the production of therapeutic proteins using transgenic plants appeared to offer a number of major advantages over other expression systems including safety (no risk of human-threatening viral contamination), low upstream costs, and complex glycosylation. Plant cell suspensions and HRCs combine the intrinsic advantages of plants and a confinement of production. In comparison to cell suspensions, HRCs present several advantages such as genotypic and phenotypic stability and possible extracellular secretion of expressed proteins (also referred to as rhizosecretion) offering a convenient method for target proteins purification in a well-defined protein-deficient medium (Wang and Wu, 2013). HRCs are capable for the production of complex compounds and high scalability (Häkkinen and Ritala, 2010; Stoger et al., 2014). In this context, the production of recombinant proteins has been considered a promising application of HRCs. It allows the expression of recombinant proteins by roots grown in bioreactor and their secretion in the culture medium under controlled and confined conditions.

Likewise, *R. rhizogenes*-mediated transformation has allowed a successful production of different chemical compounds also known as specialized metabolites or secondary metabolites which correspond to complex molecules naturally present in the plants and displaying interesting features at a pharmacological, cosmetic and nutraceutical level. According to literature analysis, over the past three decades, *R. rhizogenes* transformation has also been used to elucidate physiological processes and biosynthetic pathways, to generate plant-derived molecules, to assist molecular breeding, to improve phytoremediation strategies, and to produce therapeutic recombinant proteins (Georgiev et al., 2012; Häkkinen and Oksman-Caldentey, 2018).

**Figure 1:**
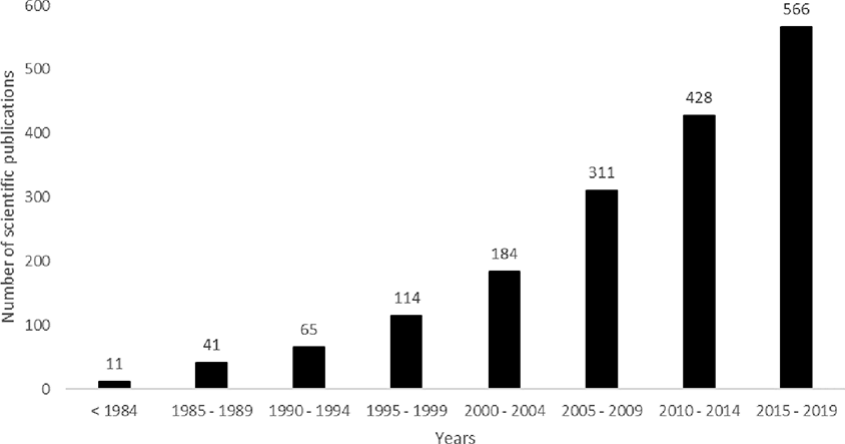
Overtime evolution of the number of scientific publications dealing with hairy roots (HRs) (date of access, December 16th, 2019 with key words “hairy roots” or “hairy root”).

Due to their technological and economic advantages, the development of HRCs has gained an increasing interest by academic research teams, biotechnology companies and pharmaceutical industries. To exemplify, according to SCOPUS databases, from 01/2012 to 11/2018, 607 articles dealt with research using HRCs. According to PubMed databases, from 01/2012 to 02/2019, 767 scientific publications were identified with the keywords “hairy roots” or “hairy root” or “transformed root cultures” or “transformed root culture.” When the terms HRCs and recombinant protein (RP) are inquired, different subject areas are identified, from which the three most relevant are Biochemistry, Genetics and Molecular Biology (38.2%), Immunology and Microbiology (17%), and Agricultural and Biological Sciences (17%). In SCOPUS database, 78 patents dealing with recombinant protein production using HRCs were identified. Most of these patents were published in 2017 and mainly comprise the description of methods to increase the production yield in plant material (Medina-Bolivar and Yang, 2017). Some are specifically dedicated to increase the secretion of recombinant proteins (Jost et al., 2014). Globally, as illustrated in **Figure 1**, the scientific research related to the HRCs is on the rise since the 1990s, with a marked increasing interest during the last 15 years (from PubMed databases).

## *R. Rhizogenes*: a Particular Bacterium

*R. rhizogenes* is a gram-negative soil bacterium inhabiting near plant roots and ultimately causing in the infected plant host the so-called “hairy root syndrome.” This syndrome consists of a non-geotropic branching root overgrowth at the site of infection (Guillon et al., 2006). The molecular events involved in the formation of the so-called HRs are not yet entirely understood. However, the genetic transformation process can be divided into the following stages: (a) *Rhizobium*-perceiving phenolic compounds e.g. acetosyringone are released by the explant usually after wounding, activating attachment of the bacteria to explant/root cells; (b) processing of the T-DNA into bacterial cells and T complex formation (T strands and other associated proteins); (c) transfer of T complexes from the bacteria to the plant host genome; (d) T-DNA incorporation and expression in the plant genome; and (e) HRs emerging from the infection site (Georgiev et al., 2012).

The T-DNA of the Ri plasmid is randomly integrated into the plant genome and expressed as mRNA. Various loci such as the so-called *vir* region of the pRi, T-DNA, and chromosomal virulent (*chv*) genes are vital for efficient transformation. These genes consist of *virD1* and *virD2*, that portray proteins that attach to and cut DNA at 25-bp T-DNA border repeat sequences (Georgiev et al., 2012). Proteins translated from *virE1* and *virE2* genes are also significant as they shield T strands from nuclease digestion and ease their integration into the plant chromosome. Even though some *R. rhizogenes* strains do not possess these genes, they still transfer T strands effectively because of the existence of the pRi *GALLS* gene portraying a protein with a nuclear localization signal and helicase activity (Gelvin, 2009). The T-DNA contains two independent sequences, namely left and right borders, TL and TR, respectively. TL-DNA and TR-DNA are usually independently transferred and stably integrated into the genome of the host plant (Chandra, 2012). However, only the TL-DNA is vital and sufficient for HR induction. After sequencing of the TL-DNA, four open reading frames were discovered as essential for HR induction (*rolA*, *rolB*, *rolC*, and *rolD*). The products of these *rol* genes have specific functions in the formation; however, the *rolB* gene seems to be the most relevant in the induction. Also the *rol*-genes have a big influence on the phenotype of the plants regenerated from the HRCs (Sevón et al., 1997). In a loss-of-function study, it was discovered that the knock-out of the *rolB* gene causes the plasmid to be avirulent (Mauro et al., 2017). Additionally, the *rolB* gene showed to be involved in RNA silencing pathways through microRNA overexpression (Bulgakov et al., 2015). Finally, the *rolB* gene of *R. rhizogenes* is involved in the activation of the transcription factors of most specialized metabolites in HRCs as well as on the expression of chaperone-type proteins (Bulgakov et al., 2016; Bulgakov et al., 2018). However, the function of this activator is still poorly understood. Very recently, a critical role of *rolA* in the long-term cultivation was also discovered, opening up a new research area to fully understand the *rol* genes function (Veremeichik et al., 2019). Overall, *rol* genes are described as modulators of plant growth and cell differentiation and could also mediate uncommon signal transduction pathways in plants (Bulgakov, 2008). Further research is still required to have a better understanding of all molecular events concerning HR induction.

## HR Process: Generation, Conservation, and Culture

The first comprehensive publication on the state of the art technology on HRCs (Hairy Roots: Culture and Applications) (Doran, 1997) provided information on laboratory protocols for initiation, culture, and genetic transformation of HRs. Additionally, the book described applications in plant propagation, alkaloid synthesis, and downstream processing considerations for large-scale HRCs. Nowadays, the laboratory protocols for HRC are highly comparable to those previously used.

To develop an optimal HRC system, the complexity of the molecules to be produced has to be considered very early in the process (such as molecular weight, potential toxicity, etc…), along with the intrinsic properties of the plant species selected for HR induction (such as growth capacity, ability to be transformed, etc…). Actually, some species are more efficient than others in terms of productivity. This is particularly true regarding the production of specialized metabolites for which the choice of the plant to infect will influence on the metabolites to be produced. For example some hemp species have to be privileged according to the cannabinoid compounds to be produced (De Meijer et al., 2003). The choice of plant species also needs to be considered for the production of recombinant proteins. As an example, one of the plant species among the most commonly used to produce recombinant proteins is *Nicotiana tabacum*. It has been demonstrated that this species is less efficient than *Brassica rapa rapa* for the production and secretion at least of eGFP under the same HRC conditions (Huet et al., 2014). Moreover, the stability over time on the production capacity of the HRs has to be considered (Huet et al., 2014; Häkkinen et al., 2016). Finally, when possible, the safety of the plant species has to be taken into account in particular for pharmaceutical application (e.g. edible plants). Globally, the two most important criteria, when selecting the most appropriate plant species for HRC, are its ability to produce and secrete high amounts of the molecule of interest and its biomass production capacity.

**Figure 2:**
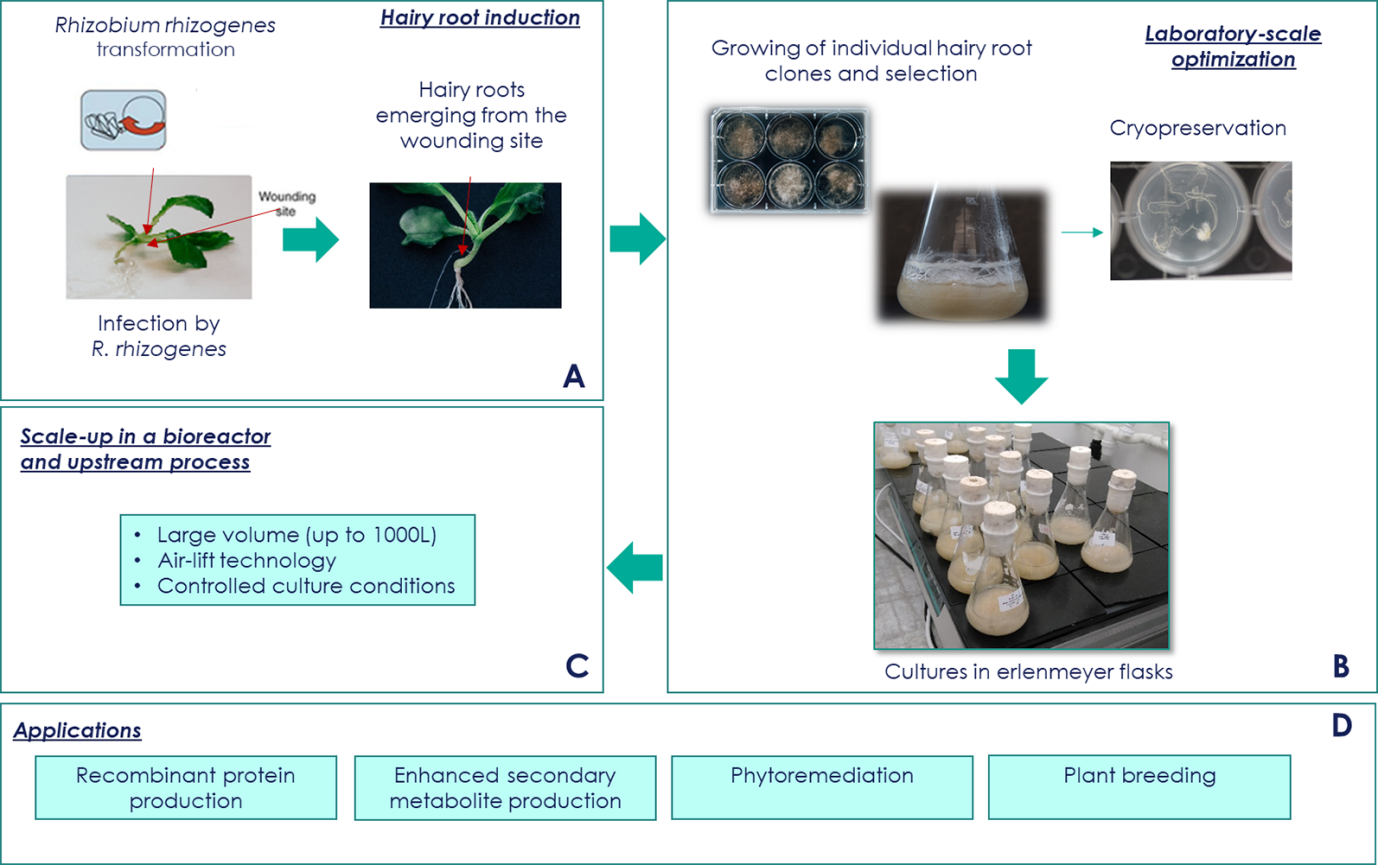
Hairy root culture platform. **(A)** Hairy root culture (HRC) induction. The efficiency of transformation depends on different factors such as type of explant and age, and the strain and density of *Rhizobium rhizogenes*. Acetosyringone addition can help to permeate the cell walls and plant membranes promoting better infection efficiency. After infection, the use ofantibiotics to eliminate excess of bacteria is necessary. **(B)** Conservation and pre-bioreactor stage. Storage clones in master/working transgenic bank is possible using cryoconservation method. In another way, several strategies can be used to improve the yields of the desired compounds. Among these strategies, there is inoculum and nutrient medium optimization, elicitation, selection of high-producing lines, metabolic engineering approaches, permeabilization, and two-phase systems cultivation. **(C)** Bioreactor stage. Different conditions can be optimized according to the desired outcome. Some of the optimizable variables are bioreactor operation mode (batch, fed-batch), culture conditions (air flow rate, temperature), hardware configurations (drive wave reactor, pneumatically driven bubble column bioreactor, and gas-phase bed reactor also referred to as “mist bioreactor”). Another medium-scale up culture approach is the use of shaken flasks using orbital shaker with HRC grown in liquid medium. **(D)** Applications. Among the possible molecules derived from HRCs there are specialized metabolites and recombinant proteins. Transgenic HRs can also be used for phytoremediation or mechanism understanding.

Usually, HRC induction involves cultivation of sterile wounded plant explants that are directly inoculated with a *R. rhizogenes* (**Figure 2A**). When the goal is to produce recombinant proteins, this bacteria have to be first genetically engineered, so as to portray the genes of interest to be later expressed by the HRs. Certain plant species, such as monocotyledonous species, are considered recalcitrant as they cannot be transformed using this method. For these recalcitrant species, a technique called sonication-assisted *R. rhizogenes*-mediated transformation (SAArT) has been demonstrated to be suitable for inducing HRCs (Georgiev et al., 2015). The explants are then treated with antibiotics to eradicate the bacteria. The resultant neoplastic HRs with multiple branches grow on hormone-free media. At this point, to confirm that these roots are indeed HRs and not adventitious roots, and that the *R. rhizogenes* was efficiently eradicated, PCR is normally performed using primers that amplify *rol* and *vir* genes. If a heterologous gene has been integrated in *R. rhizogenes* bacteria, parallel PCR using transgene-specific primers is also used in order to confirm its integration in the HR genome.

Once developed and selected, the HR strains can undergo different maintenance procedures according to financial and practical constraints (**Figure 2B**). Currently, the mainly used preservation method is a monthly subculture of individualized HRs on solid and/or liquid media. This method is time-consuming, expensive, and may present high risks of contamination and eventual loss of original strains. Another HR preservation alternative that avoids the abovementioned problems is cryopreservation of the HR clones (Georgiev et al., 2012). The cryoconservation refers to the storage of a biological material (as plant) for a long-term period in specific conditions to avoid any genetic modification or alteration that may threat the ability of the material to produce a well-characterized molecule of interest. To date, three different approaches have been proposed for the cryopreservation of plant material cultures in liquid nitrogen: (a) the slow cooling technique, (b) the vitrification method, and (c) the dehydration of immobilized cells within alginate. Few articles describe methods for cryo-conservation of plant materials and especially HRs (Hirata et al., 1998; Xue et al., 2008; Lambert et al., 2009; Häkkinen et al., 2016). These methods present advantages to be transferable in biobanks and to be in agreement with the usual definition of a Master and Working transgenic bank, thus facilitating the potential use of HRCs in GMP processes.

One of the final goals of HRCs is to produce plant-expressed compounds of interest, ideally in large-scale bioreactors (**Figure 2C**). The use of bioreactors for HRCs has to be optimized according to the species and the molecules of interest. Some of the considerations for optimization are hairiness, thickness, length, and branching of the roots. Additionally, both metabolite production and cell growth are non-homogeneous according to the plant initially transformed and to the molecule to be produced, which further complicates bioreactor optimization (Doran, 2013; Lehmann et al., 2014). Moreover, monitoring the root growth is challenging due to the inability to obtain homogeneous HR samples on a real-time basis. Furthermore, doubling time is also a crucial factor to be considered to ensure that the HR growth occurs as expected. Despite all the above-mentioned constraints, when bioreactors can be developed, they are suitable for HRCs because of their self-containment with inflow and outflow systems for liquid and air, enabling a controlled growth in sterile environments that contain only liquid nutrients. Generally, conditions such as pH, aeration, temperature, and dissolved gasses can be controlled in bioreactors (Doran, 2013).

The bioreactors used for HRCs can be classified into gas-phase or liquid-phase reactors and are generally derived from classical bioreactors but adapted for the culture of plant tissues. Also a lot of studies have been conducted using disposable bioreactors e.g. wave-type of bioreactors (Eibl and Eibl, 2008; Eibl et al., 2011; Georgiev et al., 2013). In brief, in gas-phase reactors, roots are exposed to air and other gas mixtures, and nutrients are commonly distributed to the roots as droplets of different sizes. An example of this type of bioreactor is the mist and trickle-bed bioreactors (Georgiev et al., 2013; Srikantan and Srivastava, 2018). Such bioreactor generally offers abundant oxygen supply. On the other hand, in liquid-phase reactors, roots are submerged in the medium (explaining the given name of “submerged reactors”). Examples of liquid-phase reactors are stirred tank and airlift and bubble column reactors (Doran, 2013). The advantages of this type of bioreactors are the well-known and simple design and construction, low risk of contamination, and low maintenance. Until now, most of the HRCs were grown in liquid-phase bioreactors with volume up to 20–30 L (Lee et al., 1999; Sivakumar et al., 2005; Georgiev et al., 2013). However, for some HRCs, volumes of several hundreds of liters have been reached (Wilson et al., 1990; Samabriva's internal data). The emergence of companies able to produce HRs in optimized and perfectly controlled, large volumes bioreactors, offer interesting perspectives for an industrial use of this biological material in the future.

Finally, the classical downstream process of any biosynthetic compound takes place after the production. The produced compound can be secreted into the media, facilitating the downstream processing. Nevertheless, some products may remain within the cells, thus potentially generating trouble for purification (Talano et al., 2012). It is interesting to note that sometimes non-natural or biosynthetically foreign compound can be more efficiently secreted to the culture medium than compounds which are naturally produced by the HRs. This was earlier shown by tobacco HRs overexpressing hyoscyamine-6β-hydroxylase and secreting up to 85% of the produced scopolamine in the culture medium, in contrast to the *Hyoscyamus* HRs where the majority of the product was retained in intracellular space (Häkkinen et al., 2005). Other strategies enabling a permeabilization of HRs for an optimization of the targeted metabolite/recombinant protein release into the culture medium were also used. Several approaches were tested, most of the time involving treatment with organic solvents or surfactants (Chandra and Chandra, 2011).

The products being expressed by HRs can correspond to specialized metabolites, naturally produced by the plant, or recombinant, heterologous proteins. HRs can also serve as tools for the study of gene function, phytoremediation, molecular breeding, among others (**Figure 2D**).

## Production of Recombinant Proteins

To our knowledge, the first proof of concept for HRCs was achieved by producing a mouse monoclonal antibody in HRs of tobacco plants (Wongsamuth and Doran, 1997). By then, it was shown that this antibody was secreted and accumulated in the culture medium. Afterwards, other recombinant proteins have been produced and secreted by tobacco HRs, including for example the green fluorescent protein (GFP) (Medina-Bolívar and Cramer, 2004), human acetylcholinesterase (Woods et al., 2008), murine interleukin (Liu et al., 2009), thaumatin sweetener (Pham et al., 2012), human interferon alpha-2b (Luchakivskaia et al., 2012), recombinant human EPO (rhEPO) (Gurusamy et al., 2017), and recombinant alpha-L-iduronidase (Cardon et al., 2019). Even complex glycosylated proteins can be produced using HR platforms with highly homogeneous post-translational profiles. As an example, Cardon et al. (2019) detailed, the N- and O-glycosylation profiles of a human lysosomal enzyme produced from HRC and showed that all N-glycosylation sites of this protein were occupied by paucimannose profiles with a very high homogeneity. Such paucimannosidic profile is of particular relevance for the production of proteins that could be used to treat patients with some lysosomal disorders (such as Gaucher disease or Fabry disease). Therefore, numerous heterologous proteins have been produced using HRs-based expression systems including antigens, antibodies, enzymes, and immunomodulators as described in **Table 1**.

**Table 1:** Examples of recombinant proteins produced using hairy root cultures (HRCs).

Recombinant protein	Plant species	Protein function	Culture approach	Reference
**Monoclonal antibody M12**	*Nicotiana tabacum*	Anti-cancer	Shake flasks	(Häkkinen et al., 2014)
**Green fluorescent protein (GFP)**	*Brassica rapa*	Reporter protein	Shake flasks	(Huet et al., 2014)
**Human tissue plasminogen activator (t-PA)**	*Cucumis melo*	Thrombolytic protein that converts plasminogen into plasmin	Shake flasks	(Abdoli Nasab et al., 2016)
**MAP30**	*Nicotiana tabacum*	Anti-tumor and anti-HIV protein	Shake flasks	(Moghadam et al., 2016)
**Tumor‐targeting monoclonal antibody mAb H10**	*Nicotiana benthamiana*	Anti-cancer	Shake flasks	(Lonoce et al., 2016)
**Human gastric lipase**	*Brassica rapa*	Therapeutic enzyme used for pancreatic enzyme deficiency, it contributes to fatty acid release from ingested triglycerides.	Shake flasks	(Ele Ekouna et al., 2017)
**Recombinant human EPO (rhEPO)**	*Nicotiana tabacum*	Glycoprotein hormone that influences the production of erythrocytes through erythropoiesis. EPO is used for the treatment of anemia.	Shake flasks	(Gurusamy et al., 2017)
**Virus like particle harboring recombinant protein shells of Johnson grass chlorotic stripe mosaic virus**	*Nicotiana tabacum*	Vaccine	Shake flasks	(Alemzadeh et al., 2017)
**Anti‐CD20 scFv‐Fc antibody**	*Nicotiana benthamiana*	Tumor targeting antibody	Shake flasks	(Lonoce et al., 2019)
**Alpha-L-iduronidase (IDUA)**	*Brassica rapa*	Lysosomal enzyme which catalyzes the hydrolysis of unsulfated alpha-L-iduronosidic linkages in dermatan sulfate	Bioreactors	(Cardon et al., 2019)

The design of the construct used to express the recombinant protein is a critical step. Commonly, a construct contains a promoter, a signal peptide to orientate the heterologous proteins to the expected pathway (i.e. the secretory pathway), the gene encoding the protein of interest, and a polyadenylation sequence. Gene constructs are strategically designed to contain a strong promoter for high-level gene expression. The strong constitutive promoter cauliflower mosaic virus (CaMV35s) or its enhanced version (deCaMV35S) have been most commonly used to drive transgene expression in HRCs (Georgiev et al., 2012). Besides using the above-mentioned constitutive promoters, inducible promoters can also be used. For instance, glucocorticoid- or thermo-inducible promoters are used to drive controlled gene expression at required times in HRC systems (Sun and Peebles, 2016). Moreover, the use of enhancer like TMV omega enhancer could help to improve the productivity. Retention signals such as the well-known KDEL or HDEL sequences can be incorporated in the molecular construct in order to increase the cytoplasmic content of the heterologous protein. This strategy can be relevant to prevent protein degradation in the culture medium in some situations, thus enhancing the overall production yields. For example, the use of the KDEL sequence at the C-terminal end of the 14D9 antibody increases its accumulation level in tobacco HRCs (Chahardoli et al., 2018). On the other hand, when the goal is to target the expression of a foreign protein to the secretory pathway, a dedicated signal peptide such as the ER signal peptide *cal* (N-terminal calreticulin fusion sequence) can be incorporated in the molecular construction. To illustrate such strategy this signal peptide has appeared essential for the secretion of human erythropoietin into the medium of *N. tabacum* HRCs (Gurusamy et al., 2017).

*R. rhizogenes* mediated transformation can be applied, once the molecular construct has been designed, to introduce the heterologous gene of interest in the recipient plant without changing the genetic architecture of the plant host, enabling the transgenic HR to produce the respective heterologous protein. The most common strategy consists in modifying the *R. rhizogenes* strain by integrating a heterologous gene of interest into the bacteria *via* classical molecular biology methods before infecting the plant. In this case, standard plant expression binary vectors harboring specific T-DNA with the gene of interest are used. Using this strategy, stable integration of the gene(s) of interest is obtained. Due to the random insertion into the genome of the plant, it is necessary to identify the most robust clones among all generated clones in terms of productivity and growth capacity as illustrated by Huet et al. (2014). As described in Donini and Marusic (2018) a second strategy consists in directly infecting a transgenic plant already expressing the heterologous protein using the wild type strain of *R. rhizogenes*. Thus, no genetic engineering of the bacterial strain is required. In this respect, the availability of plant mutant banks offers a wide range of possibilities.

As mentioned above, the recombinant proteins can be either produced internally or secreted into the culture medium. Large proteins– can be secreted into the culture medium of HRC (Cardon et al., 2019), enabling a large spectrum of protein production (**Table 1**). As an example, alpha-L-iduronidase, a complex human protein of 72 kDa is well-secreted into the culture medium once produced by HRCs (Cardon et al., 2019). Beyond 110 kDa, the secretion of the protein requires the use of particular strategies, such as a wall permeabilization if the protein remains bound to the biomass (e.g. using DMSO) (Talano et al., 2012). Moreover, the physicochemical characteristics of the proteins such as hydrophobicity and charge can also be important determinants for secretion and/or retention. The use of protein-stabilizing agents in the plant culture medium also represents a promising method for a stable maintenance of the productivity. Examples of protein stabilizers and protease inhibitors that can be employed are bovine serum albumin (BSA) (for protein-based stabilization), gelatine, PEG, PVP, and other polymers (to protect proteins from plant cell denaturing agents), and mannitol (to regulate osmotic pressure of the medium thus minimizing cell lysis) (Alvarez and Alvarez, 2014).

In summary, all steps of the process have to be carefully designed when producing recombinant proteins in HRCs, from the selection of the starting plant material to the molecular strategy considering not only the particularities of the heterologous gene structure itself but also its future features according to the source from where it will be purified. By meeting those criteria, the production of functional proteins within expected productivities is more likely to occur.

## Production of Specialized Metabolites

For the production of specialized metabolites, HRCs are more appropriate than cell or callus cultures due to characteristics such as genetic stability, high biomass production, and efficient biosynthetic capacity. Moreover, HRCs are able to produce specialized metabolites for a long period of time (Peebles et al., 2009; Häkkinen et al., 2016). Specialized metabolites are usually produced using non-transgenic HRCs. In this case, the plants selected are those species which naturally produce the compound of interest. Some examples of specialized metabolites already produced in wild-type HRCs are azadirachtin (biopesticide) (Thakore and Srivastava, 2017), betalain (red pigments for food industry) (Pavlov et al., 2005), camptothecin (antitumor agent in the treatment of ovarian and colorectal cancers treatment) (Wetterauer et al., 2018), and whitanolide A (brain regenerative compound) (Shajahan et al., 2017). A transgene can also be introduced in wild type HRs in order to increase the amount of a particular specialized metabolite (Jouhikainen et al., 1999; Moyano et al., 2003; Zhang et al., 2004; Ritala et al., 2014). As an example, gene silencing knock-out strategies have been used either to avoid negative regulation or to modify a biosynthetic pathway, therefore improving the production of specific compounds (Mehrotra et al., 2015). In this last case, the over- or co-expression of enzymes involved in the biosynthesis pathway may be instrumental in the production of high-value metabolites. Some examples of specialized metabolites produced using transgenic HRCs are tropane alkaloids such as scopolamine and hyoscyamine (Jouhikainen et al., 1999; Häkkinen et al., 2016; Guo et al., 2018; Khezerluo et al., 2018), catharanthine (Hu, 2006), ginsenosides (Woo et al., 2004), solanoside (Putalun et al., 2004), and vitamin C (Wevar Oller et al., 2009). Sirikantaramas and Taura (2017), utilizing transgenic HRCs and substrate feeding, were able to produce cannabinoids in *N. tabacum*, even if this species is normally not able to produce such specialized metabolite. A recent review describes some examples of valuable metabolites produced in transgenic HRs and their associated strategies, e.g. the concept of phytoremediation (Doran, 2009; Vaghari et al., 2017).

Regardless of the strategy (i.e. use of transgenic or non-transgenic HRCs), the production of specialized metabolites has been successfully improved by optimizing the growth conditions (e.g. carbon source, aeration, pH, light/dark, culture medium composition), selection of the clone (Sevón et al., 1998), and/or by paying a particular attention to the selection of adapted elicitors (Wang and Wu, 2013; Perassolo et al., 2017; Halder et al., 2018). The expression of specialized metabolites in HRCs, as in all plant-based production systems, requires the identification of the most appropriate elicitors and administration scheme. There is substantial literature exemplifying the elicitors that can be used to produce specialized metabolites using *in vitro* plant tissue culture systems (Zhou and Wu, 2006; Ramakrishna and Ravishankar, 2011; Hussain et al., 2012; Murthy et al., 2014; O'Kennedy et al., 2016). Not all of these elicitors were tested in HRCs and only a few of the most representative examples have been selected in this document to illustrate the current trend in the use of elicitors (see **Table 2**). **Table 2**, although not exhaustive, highlights the great diversity of elicitors available. However, a thorough analysis of all elicitors used in the literature shows that some candidates have been more frequently used and/or successfully tested. These include methyl-jasmonate, jasmonic acid, chitosan, or salicylic acid. The ability of an elicitor to induce a metabolic pathway is dependent on the plant and on the metabolite of interest. Also the ability of certain elicitors to affect the secretion of the metabolite has to be considered (Bais et al., 2003; Thimmaraju et al., 2003). The recognition of elicitors by the plants seems to be carried out by specific receptors on the surface of the plant cell or at the intracellular level allowing the triggering of a signal transduction cascade leading to the stimulation of the cells, a characteristic set of plant defense responses (Nürnberger, 1999).

**Table 2:** Examples of specialized metabolites produced by HRCs and examples of elicitors used.

Plant species	Products	Elicitors	References
*Ammi majus*	BION_®_	Coumarine/Furocoumarine	(Staniszewska et al., 2003)
*Arachis hypogaea*	Resveratrol, Piceatannol, Arachidin-1, and Arachidin-3	Cyclodextrin	(Yang et al., 2015)
*Astragalus membranaceus*	Isoflavonoid	Methyl jasmonate	(Gai et al., 2016)
*Brugmansia candida*	Hyoscyamine	CdCl_2_	(Pitta–Alvarez et al., 2000)
*Chicorium intybus*	Esculin/Esculetin	*Phytopthora parasitica* filtrate	(Bais et al., 2000)
*Echinacea pupurea*	Caffeic	Gibberellic acid	(Abbasi et al., 2012)
*Fagopyrum tataricum*	Rutin, quercetin	UV-B	(Huang et al., 2016)
*Hyoscyamus muticus*	Tropane alkaloids	Chitosan	(Sevón et al., 1992)
*Panax ginseng*	Ginsenoside	Methyl jasmonate	(Palazón et al., 2003)
*Papavar orientale*	Morphine	Methyl jasmonate	(Hashemi and Naghavi, 2016)
*Plumbago indica*	Plumbagin	Jasmonic acid	(Gangopadhyay et al., 2011)
*Psoralea corylifolia*	Daidzin	Jasmonate	(Zaheer et al., 2016)
*Salvia castanea*	Tanshinone	Methyl jasmonate	(Li et al., 2016)
*Salvia miltiorrhiza*,	Tanshinone	Salicylic acid	(Hao et al., 2015)
*Scopolia parviflora*	Scopolamine	Bacteria sp.	(Jung et al., 2003)
*Solanum khasianum*	Alkaloids	*Aspergillus niger*, cellulase	(Srivastava et al., 2016)

Various studies have also shown that there is a synergistic effect between several elicitors. This is the case, for example, with the cross-use of biotic and abiotic elicitors, which makes possible to increase the production of tanshinones in *Salvia miltiorrhiza* HRC (Yan et al., 2006). The increase in productivity can be moderate: 4.5-fold for the production of isoflavonoid in *Pueraria candollei* HRCs after elicitation by the yeast extract (Udomsuk et al., 2011), or can be significant as stilbenes excretion increased by 570-fold after the elicitation with MeJA and methyl-β-cyclodextrins in *Vitis vinifera* HRs (Tisserant et al., 2016). Moreover, beyond the type of elicitor, its dosage (not too strong to avoid toxicity nor too low to generate an effect), its exposure time, and the plant material and the age to which it is applied are of particular relevance.

A recently used approach for the optimization of specialized metabolites through HRCs is *in silico* modeling (e.g. fuzzy logic-based simulations, neural network). This type of computer modeling approaches has been used to optimize chemical culture conditions of HRCs (Lenk et al., 2014).

## Conclusions and Future Perspectives

In theory, HRCs can be induced from basically all plant species. Therefore, this technology could potentially be implemented to rare, valuable, threatened, or endemic medicinal species in an effort to preserve biodiversity. Moreover, HRCs have become important tools for studying biosynthesis of plant-derived molecules and they are appealing bioproduction systems with different advantages over cell suspensions and open-field-grown plants. HRCs are also very optimal model systems for recombinant protein/specialized metabolite production, or for unraveling the intricate interactions implicated in phytoremediation.

Using recent molecular biology tools, it is now possible to develop high producing clones that are able to secrete improved functional and efficient molecules for different industrial applications (food, cosmetic, or pharmaceuticals). Thus, the current state of the HRC technology should have an impact on broad commercialization of HRCs, given their several applications. This increasing interest for HRC requires developing robust cost effective GMP ready process with well-controlled systems.

Moreover, the new molecular biology tools (e.g. the CRISPR/Cas9 technology) allow a strong perspective for development of HRCs. According with PubMed Central database, the first transformation of HRs using CRISPR/Cas9 strategy has been effective in 2014, when transgenic tomato HRs producing eGFP were published (Ron et al., 2014). CRISPR/Cas9 has been used as a tool to study and optimize the mutagenesis processes in HRs of soybean (*Glycine max*) and *Medicago truncatula* (Michno et al., 2015; Du et al., 2016) and more recently for the knock-out of a gene involved in HRs architecture (BcFLA1) in *Brassica carinata* (Kirchner et al., 2017). The possibility to generate HR clones harboring knock-out gene(s) will facilitate metabolic pathway understanding or modification. This kind of experimentation can be used to reinforce specialized metabolites expression, to modify posttranslational modifications of recombinant proteins, e.g. glycosylation features or to help in the elucidation of biosynthetic pathways.

Due to an initial poor productivity and a scale-up issue, HRCs were, until now, not used at an industrial level except in the cosmetic area where an extract from a basil HRC is commercialized to treat hair loss. The strong optimization of the process in terms of production capacity, bioreactor size, and ability to modify HRCs to produce tailored-made complex molecules pave the way to a prominent place as a future biotechnology tool of this technology in plant molecular farming.

## Author Contributions

FC and AR conceived the idea of this review and designed the overall concept. FC and NGV were the most involved in the writing. SH, CL, MG, KMOC, and AR also participated in the writing of the review and/or reviewed it. All authors approved the final version.

## Funding

This review was partly supported by the European Union's Horizon 2020 Research and Innovation Program (PharmaFactory project under grant agreement No 774078).

## Conflict of Interest

FC, CL, and MG are employed by the company Samabriva SA.

The remaining authors declare that the research was conducted in the absence of any commercial or financial relationships that could be construed as a potential conflict of interest.
